# Smart Targeted Delivery Systems for Enhancing Antitumor Therapy of Active Ingredients in Traditional Chinese Medicine

**DOI:** 10.3390/molecules28165955

**Published:** 2023-08-08

**Authors:** Chenglong Kang, Jianwen Wang, Ruotong Li, Jianing Gong, Kuanrong Wang, Yuxin Wang, Zhenghua Wang, Ruzhe He, Fengyun Li

**Affiliations:** 1 School of Chinese Materia Medica, Tianjin University of Traditional Chinese Medicine, Tianjin 301617, China; kcl000804@163.com (C.K.);; 2 School of Management, Tianjin University of Traditional Chinese Medicine, Tianjin 301617, China; 3 School of Integrative Medicine, Tianjin University of Traditional Chinese Medicine, Tianjin 301617, China

**Keywords:** antitumor active ingredients, traditional Chinese medicine, nano-delivery systems, physical encapsulation, chemical coupling, pharmacological efficacy

## Abstract

As a therapeutic tool inherited for thousands of years, traditional Chinese medicine (TCM) exhibits superiority in tumor therapy. The antitumor active components of TCM not only have multi-target treatment modes but can also synergistically interfere with tumor growth compared to traditional chemotherapeutics. However, most antitumor active components of TCM have the characteristics of poor solubility, high toxicity, and side effects, which are often limited in clinical application. In recent years, delivering the antitumor active components of TCM by nanosystems has been a promising field. The advantages of nano-delivery systems include improved water solubility, targeting efficiency, enhanced stability in vivo, and controlled release drugs, which can achieve higher drug-delivery efficiency and bioavailability. According to the method of drug loading on nanocarriers, nano-delivery systems can be categorized into two types, including physically encapsulated nanoplatforms and chemically coupled drug-delivery platforms. In this review, two nano-delivery approaches are considered, namely physical encapsulation and chemical coupling, both commonly used to deliver antitumor active components of TCM, and we summarized the advantages and limitations of different types of nano-delivery systems. Meanwhile, the clinical applications and potential toxicity of nano-delivery systems and the future development and challenges of these nano-delivery systems are also discussed, aiming to lay the foundation for the development and practical application of nano-delivery systems of TCM in clinical settings.

## 1. Introduction

**Figure 1:**
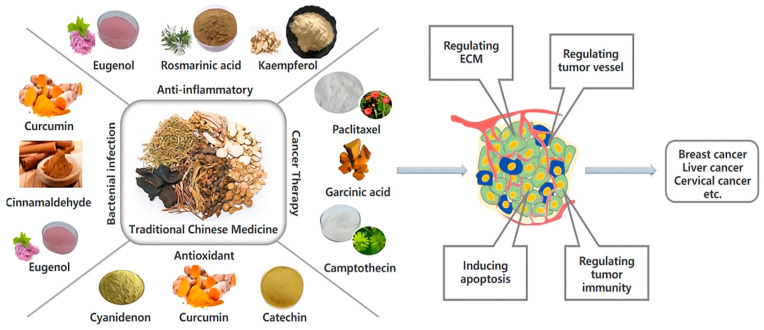
The application of active components of traditional Chinese medicine in biomedicine.

Malignant tumors are a major threat to human health. According to the Global Cancer Report released by the International Agency for Research on Cancer (IARC), the cancer incidence in the world has gradually increased, and the majority of malignant tumors are already in the middle or late stages when they are diagnosed [1]. Typical clinical treatments include surgery, radiotherapy, and drug therapy (also called “chemotherapy”). Chemotherapy is more suitable for some tumors with systemic dissemination tendency and patients with metastatic mid to late-stage tumors compared to surgery and radiotherapy, which can only target localized tumors [2,3]. However, most chemotherapy components have strong toxic and side effects, and long-term usage is also prone to drug resistance, which leads to the reduction of therapeutic effect. Traditional Chinese Medicine (TCM), an essential part of modern medicine, also plays a vital role in antitumor therapy recently. With the advancement of isolation and purification technology and in-depth research on drug mechanisms, the active ingredients of traditional Chinese medicine (such as curcumin, gambogic acid, and glycyrrhizic acid) have become more prominent in tumor therapy (Figure 1) [4]. Compared with synthetic chemotherapeutics, natural active ingredients of TCM have many unique advantages including synergistically modulating multiple tissue and molecular targets simultaneously, reducing toxic side effects, and overcoming drug resistance, which can enhance the therapeutic efficiency [5]. Although the active ingredients of TCM exhibit promising antitumor ability, the clinical application of TCM is still limited. First, most active ingredients have poor solubility and tumor-targeted capability, which leads to low bioavailability and reduced tumor-targeting efficiency in vivo delivery [6]. Second, some active ingredients of TCM have poor stability and photosensitive properties that are easily oxidized, hydrolyzed, or isomerized [7,8]. Third, certain active ingredients of TCM possess large molecular weights and exhibit weak lipophilicity, resulting in low transport efficiency and poor permeability [8]. Fourth, the high clearance rate and short half-life in vivo reduce the drug concentration in tumor tissues and are unable to achieve effective pharmacological activities, thereby weakening therapeutic outcomes [9].

**Figure 2:**
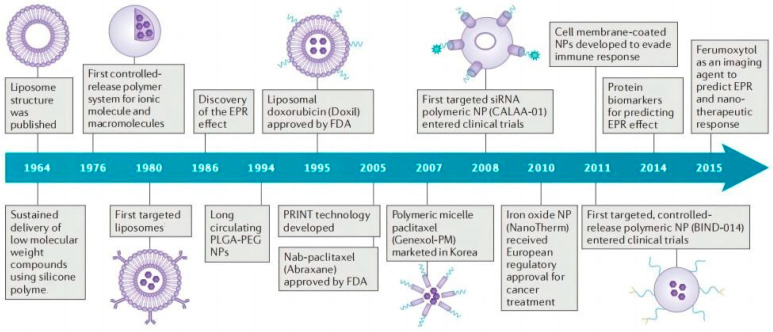
The timeline of main developments of nano-delivery system. Adapted with permission from Ref. [12]. Copyright 2016, Springer Nature.

Nano-delivery systems have emerged as indispensable tools to improve a series of problems existing in the delivery of antitumor active ingredients of TCM. As early as the end of the 1960s, liposomes were used for drug delivery because of their good biocompatibility and low cytotoxicity [10,11]. In order to achieve stronger drug-loading ability and stability, other types of nano-delivery systems have been developed (Figure 2), such as polymer nanoparticles, micelles, nanogels, etc. [12]. These nanoplatforms primarily realize the targeted delivery of active ingredients of TCM via two mechanisms: Passive targeting through the enhanced permeation and retention effect (EPR effect) and active targeting mediated by ligand–receptor recognition. Ultimately, these systems achieve smart targeted delivery and responsive drug release while minimizing the toxic side effects of active ingredients on healthy organs [8]. Furthermore, delivering active ingredients of TCM by nano-delivery systems is expected to overcome limitations associated with traditional therapeutic approaches. Several nano-delivery systems with the loading of glycyrrhetinic acid, curcumin, quercetin, rhamnetin, and resveratrol have been successfully utilized for antitumor treatment [13], which possess better solubility, bioavailability, and specific targeting compared with free drug.

Currently, nanocarriers loaded with antitumor active ingredients of TCM are primarily categorized into two methods, including physical encapsulation and chemical coupling. Physical encapsulation is one of the main drug-loaded methods, in which the drug is encapsulated in the nanocarrier core through physical interaction between the drug and the carrier [14]. Due to the simplicity of physical encapsulation and the fact that the structural and property requirements of the encapsulated drug are not stringent, this method is usually chosen for loading drugs. Depending on the different types of nanocarrier loading active ingredients of TCM, physically encapsulated nano-delivery systems can be broadly categorized into lipid nanosystems, polymeric nanosystems, metallic nanosystems, and miscellaneous approaches. However, the loading stability of physically encapsulated nanoparticles has certain limitations in that unavoidable drug leakage occurs in blood circulation leading to reduced drug efficacy. In order to precisely control the drug release, environmental response primitives can be introduced into the nanosystem to design a smart stimulus-responsive encapsulated drug-delivery system, so that the nanoplatform can remain stable during blood circulation and recognize subtle environmental changes and then respond in order to deliver drugs to specific locations.

Chemical coupling, which refers to the covalent coupling of drug molecules to the surface or interior of nanocarriers via chemical bonds, can also significantly increase the stability of drug delivery in vivo [15]. Of note, drug coupling via stable chemical bonds may be difficult to release resulting in ineffective drug release. In order to achieve controlled drug release, tumor microenvironment-sensitive chemocoupling pro-drug systems can be designed according to the unique characteristics of the tumor microenvironment, such as weakly acidic pH, hypoxia, etc., which can trigger drug release in the tumor microenvironment, further reduce drug leakage, and improve delivery efficiency.

In this paper, we focused on summarizing two kinds of smart targeted nano-delivery systems within the last 20 years, encapsulated drug-delivery systems and covalently bound pro-drug systems, designed based on physical encapsulation and chemical coupling for the efficient delivery of antitumor active ingredients of TCM, and discussed the advantages, prospects, and limitations in the clinical application of different nano-delivery systems, aiming to provide new insights for developing more efficient delivery systems for the antitumor active ingredients of TCM.

## 2. Methodology

Based on the academic journals included in Baidu Scholar, X-MoL Academic Platform, PubMed, and Web of Science (WOS) from 2003 to 2023, the literature related to the use of nano-delivery systems for delivering anti-tumor active ingredients of traditional Chinese medicines was searched for the search terms “nano delivery”, “traditional Chinese medicine”, “antitumor therapy”, and “active ingredients”. The searched literature was integrated using Endnote X9 software, and after weight removal, it was included in the final literature and exported in “RefMan (RIS) Export” format.

## 3. Nano-Delivery Systems Loaded with Antitumor Active Ingredients of TCM

### 3.1. Lipid Nano-Delivery System

Lipid nano-delivery systems offer numerous benefits, including increased retention time of active ingredients in the body, improved targeting efficiency, and enhanced solubility of Chinese medicine [16,17,18]. These systems can be categorized into liposomes, solid lipid nanoparticles, nano-lipid structured carriers, microemulsions, and self-micro emulsifying drug-delivery systems.

#### 3.1.1. Liposomes

Liposomes are small vesicles composed of lipid bilayers, which closely resemble biological membranes. Based on the special structure of liposomes, the antitumor active components of TCM with poor water solubility can be encapsulated in liposomes, thus improving the bioavailability of drugs. For instance, Zheng et al. [19,20,21] integrated 9-nitro camptothecin, which has poor solubility and stability and low oral bioavailability, into liposomes by thin-film ultrasound technology. The liposome formulation plays a significant antitumor role by inducing cell cycle arrest and apoptosis while minimizing toxic side effects on healthy tissue. In addition to increasing the solubility of drugs, liposomes can also prolong the survival time of antitumor active ingredients of TCM in vivo [22]. For example, Tang et al. [23] prepared glycyrrhetinic acid nanoliposomes with the optimized size of 75 nm (drug: liposome = 1:5). Compared to free gambogic acid, the circulating half-life of the liposomal formulation was prolonged by 17.1 h, and its antitumor activity also increased. Similarly, Wang et al. [24] constructed a novel reactive oxygen species (ROS)-activated liposomal nano-platform by loading ROS-sensitive paclitaxel (PTX) derivatives (PSNs) into liposomes. This approach overcame the main challenges associated with premature drug release and limited accumulation of PTX in tumors. Compared with free PTX, the half-life of liposomes combined with PSNs in in vivo circulation was increased nearly 100 times, which had a good antitumor effect, and the survival rate of tumor-bearing mice was prolonged more than 80 times, which can be regarded as an efficient and low-toxic delivery method. In addition, liposomes also offer other benefits: (1) Altering the pharmacokinetics and biodistribution of antitumor active components of herbal medicines, reducing toxicity, and improving therapeutic indices [25]. For example, Chen et al. [26,27] prepared strychnine liposomes to enhance the antitumor activity and reduce the influence on the central nervous system. Additionally, Xin et al. [28] developed a brain tumor-targeted liposome (RVG15-Lipo) using RVG15 rescreened by RVG29, which could improve blood–brain barrier permeability and realize tumor-specific delivery of PTX. This liposome exhibited high encapsulation efficiency (EE) and good stability and contributed to promoting high accumulation of antitumor agents in glioma, thereby significantly inhibiting glioma growth and metastasis and improving survival rates in tumor-bearing mice. (2) Liposomes also achieve drug co-delivery and synergistic effects. Wang et al. [29] designed a liposomal nanocarrier using a thin-film hydrophobic method that co-encapsulated the autophagy inhibitor hydroxychloroquine (HCQ) and PTX to effectively inhibit tumor growth. Li et al. [30] developed a nanocomposite (AuNS@ZrTCPP-GA, AZG) based on glycyrrhetinic acid (GA), gold nanostar (AuNS), tetra (4-carboxyphenyl) porphyrin (TCPP), and Zr^4+^. PEGylated liposomes were used to encapsulate AZG to improve its stability and biocompatibility. GA acted as an inhibitor of heat shock protein 90 (HSP90), while AuNS acted as a photothermal reagent. After the nanoplatform was internalized by tumor cells, AZG was slowly degraded in the weak acidic tumor microenvironment, releasing its components and exerting synergistic therapeutic effects through AuNS-mediated mild photothermal therapy (PTT) and TCPP-mediated photodynamic therapy (PDT). These synergistic effects could further induce more potent secondary necrosis/advanced apoptotic cells and enhanced antitumor effects. (3) Furthermore, liposomes target tumors to achieve precise delivery. Zhu et al. [31] reported Rg3-based liposomes prepared by thin-film hydration and loaded with PTX to target delivery to breast cancer cells, achieving a high tumor suppression rate of 90.3% via the dual antitumor effect of recognizing GLUT-1 and inhibiting p-STAT3 pathway activation. Dang et al. [32] prepared nuclear peptide CB5005N-modified CB5005n-ga-liposome via the thin-film water method to reduce the off-target limitations of Gambogic acid (GA) and promote its antitumor activity. This liposome delivery system enabled active targeted drug delivery by targeting specific targeted peptides (CB5005N) attached to the surface of tumor cells or tumor-associated stromal cells. In vivo antitumor efficacy and a biological distribution assay in female BALB/c mice showed that, compared with free GA groups, CB5005N-GA-liposome had better targeting effects and antitumor efficiency. (4) Lastly, liposomes can be used as an immune adjuvant to promote the immune activity and antitumor effect of the antitumor active ingredients of TCM. Wei et al. [33] utilized a lipid adjuvant to increase the bioavailability of ginsenoside Rg3 in a TCM formulation, resulting in enhanced antitumor activity by stimulating immune cells and inducing cytokine production. Andy et al. [34] developed a multifunctional liposome (PTX/CALP) based on the original corosolic acid liposome (CALP) vector, which inhibited immunosuppression in tumor tissues, improved the immunosuppressive microenvironment, and enhanced the ability of chemotherapeutic agents to induce immunogenic cell death (ICD). CALP had a greater cellular internalization ability and higher tumor inhibition rate compared to PTX liposomes prepared from cholesterol and phospholipids (PTX/LP). Moreover, this multifunctional liposome could be easily prepared using common liposome techniques without further modification, which was considered a simple and efficient delivery strategy.

Overall, liposomes are widely used because of their good biocompatibility and low toxicity. However, liposomes are still limited in practical production and application due to their unstable structure and susceptibility to hydrolysis and drug leakage in the process of delivering active ingredients of TCM. Moreover, liposomes are and easily and quickly removed in the blood circulation, which affects the delivery efficiency. Currently, strategies to modify the surface of liposomes are primarily used to avoid liposome recognition by the reticuloendothelial system (RES) and thus prolong circulation time. Common approaches include modifying chitosan or polyethylene glycol on the surface of liposomes or utilizing cell membranes to encapsulate liposomes to improve liposome utilization [35,36,37].

#### 3.1.2. Solid Lipid Nanoparticles (SLNs)

Solid lipid nanoparticles are solid gel drug-delivery systems made of natural lipids or synthetic lipids, usually ranging in size from 10 to 1000 nm, which can encapsulate or embed drugs in lipid-like cores to achieve targeted delivery and controlled drug release [38]. Therefore, SLNs are promising carriers for delivering antitumor active components in TCM.

SLNs loading the antitumor active ingredients of TCM have the following advantages: (1) Improves drug solubility and stability. For example, Curcumin, as an active ingredient in Curcuma longa, has many pharmacological activities, such as anti-inflammatory and antitumor, but its poor solubility and stability limit clinical application. Therefore, Wang et al. used SLNs to load CUR to increase the therapeutic efficiency of CUR [39]. The prepared CUR-SLNs had a spherical structure of 40 nm, and the encapsulation efficiency reached 72.24%. Of note, SLN delivery could significantly increase the uptake ability of CUR by SKBR3 cells, which had stronger green fluorescence in tumor cells. In addition, the toxicity of CUR-SLNs was relatively increased, primarily because Cur-SLNs promoted the ratio of Bax/Bcl-2, reduced the expression of cyclin D1 and CDK4, and then induced apoptosis and cell cycle arrest. Treponema pallidum (CPT) is a potent antitumor agent with poor solubility and instability of the active lactone form. To enhance the in vivo antitumor effects of CPT, loading CPT into structurally stabilized SLNs was a promising delivery strategy, which not only improved the water solubility of the drug but also prolonged the blood circulation of CPT in vivo [40]. Compared with free CPT, CPT-SLNs increased plasma CPT levels, and the drug concentration at the tumor site was significantly higher than in other organs. In addition, to reduce the recognition and clearance of CPT-SLNs by RES, Jang et al. pre-injected blank SLNs before administering CPT-SLNs to the tumor, thus reducing the accumulation of CPT-SLNs in the organs of RES, which was effective in the in vivo anti-tumor experiments, with a tumor volume of only 2295 mm^3^. (2) SLNs also improve drug bioavailability. Samira et al. [41] designed SN38-loaded SLNs and evaluated the drug uptake efficiency in vitro by Caco-2 and C26 cell lines. It was observed that SN38-loaded SLNs were significantly internalized in C26 and Caco-2 cell lines at 2 and 4 h compared to free SN38. (3) SLNs also enable the co-delivery of dual drugs. For instance, PTX and curcumin (CU) were co-delivered by SLNs [42] for the treatment of lung cancer. These codelivery nanosystems demonstrated a remarkable tumor inhibition rate of 78.42% in a nude mouse transplantation tumor model, which was significantly higher than the tumor inhibition rates of PTX (40.53%) and (CU + PTX) (51.56%). Moreover, these SLNs were also capable of inhibiting glycoprotein efflux, inhibiting the NF-κB pathway, and reversing multidrug resistance.

In conclusion, SLNs are characterized by greater stability and biocompatibility and low susceptibility to erosion compared to liposomes. However, SLNs still have some limitations, including poor stability during storage, particle size growth, or drug degradation. In addition, it is easy to form a structure with perfect crystals and reduce the space for drug loading [43,44,45]. To address these limitations of SLNs, researchers have explored nanostructured lipid carriers as a potential solution.

#### 3.1.3. Nanostructured Lipid Carrier (NLCs)

NLCs, the second generation of lipid nanoparticles, are developed from a mixture of solid and liquid lipids [46]. NLCs show prolonged blood circulation time, higher stability, and controlled drug release, leading to enhanced therapeutic efficacy [47]. For instance, Marathe et al. [48] developed PTX-loaded NLCs using α-tocopheryl succinate (αTS) with a uniform spherical structure, and the encapsulation efficiency was higher than 95%. These NLCs had great stability and drug release ability, which remained stable for 60 days under refrigerated conditions, and 67% drug release within 48 h. However, this study only carried out basic research on NLCs loaded with PTX and did not investigate the antitumor effect of this system in vitro. Yuan et al. [49] prepared cell-penetrating peptide-coated and tripterine-loaded lipid carriers (CT-NLCs), which significantly enhanced the antitumor activity of prostate tumor cells in vitro compared to tripterine-free drugs, and it was dose-dependent. In in vivo tumor inhibition experiments, the inhibition rates of high-dose and low-dose T-NLCs could reach 72.68% and 54.50%, respectively, which obviously increased the therapeutic effect of active components of TCM on prostate cancer. Akanda et al. prepared NLCs for the delivery of curcumin (CRN) by high-pressure homogenization for the treatment of prostate cancer. Cellular uptake results demonstrated that this delivery system could be effectively internalized by PANC-1 cells, showing strong green fluorescence in the cytoplasm. Flow cytometry analysis exhibited that CRN-NLC was able to induce apoptosis in 76.9% of the cells. In in vivo studies, these nanoparticles also displayed potent tumor suppressive effects, making them a suitable delivery system for the treatment of prostate cancer [50]. Similarly, Dolatabadi et al. also encapsulated CUR into NLCs to form a nano-delivery system, and the encapsulation rate could reach 97.37% ± 2.83 [51]. Importantly, CUR-NLC was able to significantly improve the pharmacokinetic profile of the drug in vivo, and its maximal plasma concentration was 3.41-fold higher than that of free CUR, and the area under the curve (AUC) values also increased by 3.342, providing a reference for the in vivo application of CUR. In addition, NLCs can be regarded as codelivery systems. Lages et al. [52] used NLCs to codeliver paclitaxel (DTX), dolphin fish oil, and α-tocopheryl succinate and evaluated in vitro for tumor cell toxicity. The drugs encapsulated in NCLs significantly inhibited tumor growth, reduced mortality in mice, prevented lung metastasis, and reduced DOX-induced cardiac and hepatic toxicity.

In summary, compared with SLNs, NLCs add liquid lipid materials into solid lipid carrier materials, which increases the degree of crystal disorder of carrier materials, increases the loading space of drugs, and reduces drug leakage [53,54]. Therefore, NLCs have an improved drug-carrying capacity and are less likely to have drug leakage during storage. NLCs also face some challenges in delivering the antitumor-active ingredients of TCM. These challenges include low encapsulation rates for hydrophilic antitumor ingredients, conflicts between drug loading and slow-release performance, leakage of antitumor ingredients, and burst-release phenomena.

#### 3.1.4. Microemulsion and Self-Micro Emulsion Drug-Delivery System

Microemulsions are thermodynamically stable liquid solutions that contain oil, water, and amphiphilic substances. Typically, a microemulsion consists of four components: The water phase, oil phase, surfactant, and co-surfactant. Microemulsions can be classified into three types: Oil-in-water (O/W), water-in-oil (W/O), and bicontinuous. The use of microemulsions to load insoluble anti-tumor active ingredients of TCM can enhance drug solubility, bioavailability, and anti-tumor activity [47]. Abbas Rahdar et al. [55] synthesized F127-based Curcumin (CU) microemulsions with a high encapsulation rate and long drug release time. According to in vitro studies, CU microemulsion was twice as potent as free CU at killing MCF-7 cells. Self-micro emulsifying drug-delivery systems typically consist of an oil phase, surfactant, and co-surfactant. Such systems can form microemulsions spontaneously in a suitable environment (37 °C, aqueous phase, light agitation). Due to the small diameter and large surface area, the tumor cell uptake rate of drugs, dissolution, and release can be increased, thus improving bioavailability [56]. Lin et al. [57] developed a hybrid self-micro emulsifying drug-delivery system co-loaded with a CU phospholipid complex and NIR dye to achieve the inhibition of breast cancer lung metastasis via combined chemotherapy and phototherapy. This delivery system improved the uptake of Caco-2 cells, increased the bioavailability of CU in rats, enhanced the cytotoxicity of 4T1 cells, and inhibited their migration and invasion by affecting the NF-κB pathway.

There are many advantages of microemulsion and self-microemulsion drug-delivery systems, including increasing the solubility of drugs, avoiding direct contact between drugs and the peripheral environment, protecting drugs from being degraded by active substances in vivo, enhancing drug stability, and reducing drug irritation at the same time, and has broad application prospects [58,59]. However, there are few clinical applications at present, which may be due to the need to use a large number of surfactants when forming microemulsion, which has certain toxicity, so there is no obvious progress in the research. In addition, their clinical application is still limited due to the large amount of surfactant and co-surfactant required for preparation, which can cause gastrointestinal irritation and other adverse effects [13].

In addition to the applications mentioned above, there are many other examples of lipid carriers that can be loaded with TCM’s antitumor components. These examples are listed in Table 1.

### 3.2. Polymeric Nano-Delivery Systems

The polymer nano-delivery system is an emerging delivery method with significant stability and payload. Compared with the lipid delivery system, this delivery system can not only improve the pharmacokinetic and pharmacodynamic properties of Chinese traditional medicine anti-tumor active ingredients but also has the characteristics of easy modification and biodegradation. According to the different types of delivery carriers, polymer nano-delivery systems are divided into polymer micelles, polymer nanoparticles, dendrimer macromolecules, and biopolymer-based nanocarriers.

#### 3.2.1. Polymer Micelles

Polymer micelles are a new rapidly developing nano-delivery system, which is primarily formed by the self-assembly of amphiphilic block copolymers in aqueous solutions. The antitumor active components of TCM can be effectively loaded into the hydrophobic core of micelles, thus improving the efficiency of drug delivery. For example, baicalin, as the main component of TCM scutellaria baicalensis, has many advantages such as inhibiting the proliferation and metastasis of tumor cells, but its poor water solubility and low bioavailability limit its clinical application. To this end, Zhang et al. prepared a baicalin-loaded mixed micelle delivery system (BC-ST-P123-MMs) using sodium taurine (ST) and P123 block copolymers as amphiphilic molecules by the thin-film dispersion method. The delivery system can not only improve the solubility of baicalin (10.20 mg/mL) but also improve the inhibitory effect of baicalin on HepG2 cells. Compared with free baicalin, the IC50 of BC-ST-P123-MMS was reduced to 46.18 ug/mL [74].

Paclitaxel is a Chinese medicine active ingredient with poor water solubility and a lack of targeted properties, which can interact with microtubule polymers and inhibit tubulin dissociation, thus promoting cell apoptosis. Li et al. [75] designed a pH-sensitive polymer micelle based on PEG-TPP for the delivery of PTX. After the PTX-micelle was endocytosed into tumor cells, the hydrazine bond in the PEG-TPP structure was broken in a weakly acidic environment to achieve responsive drug release and reduce the toxic side effects caused by the non-specific release of PTX. By observing the tissue distribution of the green fluorescence of PEG-TPP in vivo, it was found that the micelle had good tumor targeting, and only a strong fluorescence signal was detected in the tumor. An in vivo anti-tumor study using U87MG tumor-bearing mice as a model showed that compared with free PTX (1600 mm^3^), PTX-loaded micelles had an obvious tumor inhibition effect, a tumor volume of approximately 1000 mm^3^, the lowest systemic toxicity, and a survival rate after 14 days of 100%. The micelle not only overcame the disadvantage of the premature release of drugs but also achieved high efficiency and low toxicity anti-tumor effects, making it a promising nano-delivery platform.

It is worth noting that polymer micelles can not only increase the solubility of the antitumor active ingredients of TCM but also the hydrophilic shell of the micelles can avoid the recognition and clearance of the reticuloendothelial system and prolong the blood circulation time of the drugs. For example, Liu et al. [76] used mPEG-b-PHEMA-5HA as a monomer and CUR as a crosslinking agent to form pH-sensitive reversible crosslinked micelles via the phenol-acetylene click reaction. Compared with non-crosslinked micelles, the crosslinked micelles had stronger stability and higher drug loading (17.81%). When the micelles were delivered to tumor cells by the EPR effect, the ethylene-ether bond cleavage in the phenol-acetylene click product at weakly acidic pH led to the release of CUR. CLSM images showed that the micelle was able to be effectively endoannexed by cells to release CUR, and significant yellow fluorescence was observed in Hela and 4T1 cells.

Importantly, the negative surface potential of the micelles is easier to evade recognition by the endothelial reticular system, and pharmacokinetic studies have shown that the cross-linked micelles can effectively prolong the half-life of the CUR (6.16 h), which is 6 times that of the free CUR. The novel design of the delivery system does not simply load the anti-tumor active ingredients of TCM inside the micelles, but forms cross-linked micelles through click chemistry that efficiently load drugs and achieves responsive drug release, effectively prolongs the blood circulation time of CUR in vivo, and greatly increases the bioavailability of drugs. It is a new polymer delivery system with good application prospects.

In general, polymer micelles are designed to be more flexible by modifying targeted ligands on the polymer chain, including monoclonal antibodies, nucleic acid aptamers, or cell-specific peptides, or modifying responsive chemical bonds such as hydrazone bonds, disulfide bonds, TK bonds, etc., which endows polymer micelles with the ability to target and control drug release [77]. Although polymer micelles with multiple functions have been designed, polymer micelles containing traditional Chinese medicine anti-tumor active ingredients are still in the laboratory stage, primarily because the toxicological pharmacological properties of the embedded traditional Chinese medicine anti-tumor active ingredients are not fully clear, there are few polymer exciparants to choose from, and the cost of nano-transformation is too high.

In conclusion, compared with lipid nano-delivery, the design of polymer micelles is more flexible, and polymer micelles can be modified in different structures according to the requirements of the delivery system, thus achieving the ability of targeted or controlled drug release [78,79]. In addition, the hydrophilic outer layer of polymer micelle can effectively avoid the removal of RES and prolong the blood circulation time, which has good application prospects. However, polymer micelles lack the ability to carry water-soluble drugs, and can only carry insoluble active ingredients of traditional Chinese medicine to improve the solubility and stability of drugs.

#### 3.2.2. Polymer Nanoparticles

Polymer nanoparticles, also known as nanospheres, are solid particles with a particle size of 1–100 nm composed of polymer materials that can encapsulate the active ingredient within their core or adsorb it on their surface. These nanoparticles have several advantages:

(1) Achieve responsive drug release, thereby increasing its therapeutic effect [58,59]. For example, He et al. [80] synthesized a polymer nanoparticle self-assembled by photoactivated metal polymer polymerization (Ru/PTX), where the photosensitizer Ru complex and PTX can attach to the polymer network and be delivered simultaneously to the tumor site through EPR effects. Singlet oxygen (^1^O_2_) produced by the photosensitizer after red light irradiation further triggers the release of PTX to achieve the combination of photodynamic and chemotherapy therapy. The results showed that the light-triggered cascaded drug release polymer nanoplatform could effectively reduce the non-specific release of drugs, and the tumor growth inhibition rate reached approximately 65%. In an in vivo antitumor study, 4T1 tumor-bearing mice could induce the photolysis of Poly (Ru/PTX) after local illumination, promote the release of PTX, and exert an anti-tumor effect, which is a feasible on-demand drug delivery strategy.
(2) Polylactic acid, polyglycolic acid, and their copolymer poly (propylene glycol glycolic acid) (PLGA) are widely used due to their stability and controlled drug release. Snima et al. [81] prepared silymarin-supported PLGA nanoparticles (SNPs) by emulsifying solvent volatilization. The nanoparticle not only has good serum stability and blood compatibility, but also has good loading (the encapsulation rate is 60%) and drug-release ability. The results showed that the drug release was slow and continuous under physiological conditions, and the release rate reached 78% at 120 h. In vitro experiments demonstrated the potential of this nanoparticle in the treatment of prostate cancer. Flow results showed that SNPs could induce 63.6% apoptosis of PC-3 cells and inhibit tumor cell migration. Although PLGA-based nanoparticles show strong therapeutic effects in in vitro studies, high doses of organic materials during delivery in vivo may increase toxicity, and nanoparticles are easily recognized and cleared by the immune system. The delivery efficiency of solid tumors is greatly reduced [82]. Therefore, in order to improve the delivery effect of nanoparticles in vivo, Song et al. [83] developed a PH-sensitive bionic drug-delivery system (FRCS NPs) covered by erythrocyte membranes. The nanoparticle has an exquisite core–shell structure, and the core nanoparticle is formed from a natural polymer, sodium carboxymethyl cellulose, and stearic acid, which are self-assembled. Sodium carboxymethylcellulose is highly sensitive to the weakly acidic pH of the tumor microenvironment and promotes the responsive release of PTX. The nanoparticles coated with DSPE-polyethylene glycol (PEG) -FA-modified erythrocyte membrane can extend the cycle time in vivo and enhance the tumor-targeting ability. By observing the biological distribution of FRCS NPs in vivo, it was found that the fluorescence signal was strongest in tumors, while there was almost no fluorescence signal in organs such as the liver. Compared with free PTX, xenograft HepG_2_ tumors were effectively suppressed after caudal vein injection of FRCS NPs, and the inhibition rate reached 48.6%. In addition, FRCS NPs also have good safety in vivo, essentially have no effect on the liver function of mice, and can also reduce PTX-induced renal toxicity.
(3) Polymeric nanoparticles can be easily modified to develop various functional nanoparticles. For instance, in order to enhance the antitumor effect, researchers investigated the use of dongle-in (ORI) poly (D, L-lactic acid) (PLA) nanoparticles [84]. Additionally, PLA nanoparticles were modified by arginine-glycine-aspartate peptide (RGD) to enhance H22 cells targeting. After dosing in tumor-bearing mice, RGD-modified RGD-ORI-PLA nanoparticles exhibited higher antitumor activity compared to non-targeted ORI-PLA-NPs.
(4) Polymer nanoparticles can achieve dual-drug codelivery with drugs with different anticancer mechanisms. Due to the complexity of tumor formation, the chemotherapy effect of a single drug may be limited, so the simultaneous delivery of more than two drugs targeting different anti-cancer pathways is important to improve anti-cancer efficacy and reduce side effects. For example, Dox is a commonly used chemotherapy drug for the treatment of advanced liver cancer, which can cause DNA damage and promote apoptosis. CUR, as a common anti-tumor active component of TCM, not only has anti-angiogenesis activity but also has high safety. However, the physicochemical properties and pharmacokinetics of these two drugs are different, and it is difficult to achieve combined administration. Therefore, Zhang et al. [85] prepared a pH-sensitive polymer-based D-A-tophenol polyethylene glycol 1000 block poly (B-aminoester) (TPGS-PAE) self-assembled nanoparticle (D + C)/NPs for co-delivery of Dox and Cur, which can quickly release drugs into the acidic environment of cancer cells to achieve two-drug synergistic therapy. In vitro studies showed that the cytotoxicity of dual-drug co-delivery micelles was better than that of free single-drug micelles and showed stronger pro-apoptotic activity (an apoptosis rate of 76.2%). In addition, the total amount of Akt, mTOR, Erk, and FAK in D + C/NP-treated cells did not change, which proved that D + C/NPs could achieve an anti-angiogenesis effect by inhibiting the pathway induced by VEGF and is a dual-drug co-delivery platform with good application prospects. In the process of co-delivery, enhancing the targeting of nanoparticles is helpful to achieve accurate delivery of dual drugs. Kim et al. coupled the integrin ring (arginine-glycine-aspartic acid-phenylalanine-lysine) (cRGDfK) with active targeting and sulfonyl cyanide 5.5 (Cy5.5) with PEG-PLGA, respectively. Functionalized polymer nanoparticles based on polyethylene glycol -PLGA were formed via self-assembly for the targeted co-delivery of CUR and PTX to breast cancer [86]. The existence of cRGDfK significantly enhanced the uptake of nanoparticles by tumor cells, and CLSM images showed that the dual-drug co-delivery system had stronger red fluorescence in 4T1 cells, with a fluorescence intensity 1.83-fold that of passively targeted NPs. In vivo imaging showed that the fluorescence intensity of Cy5.5-cRGDfK-NPs/PTX + CUR was the highest and obviously concentrated at the tumor site, which also indicated that cRGDfK had a high affinity for integrins on the surface of 4T1 cells. Of note, CUR as a P-gp inhibitor combined with PTX can reverse the resistance of breast cancer to PTX and enhance the therapeutic effect. Compared with free PTX and CUR, the tumor volume of mice treated with dual-drug co-delivery nanoparticles for 18 days was only 400 mm^3^. Overall, the Cy5.5-cRGDfK-NPs/PTX + CUR showed good therapeutic potential for breast cancer.

In brief, polymer nanoparticles can better penetrate the cell membrane and deliver drugs to the lesion site because of their small size and high specific surface area, thus reducing the toxic and side effects of drugs and improving the curative effect [87]. However, the nanoparticles may be toxic and easy to aggregate, so further optimizing the composition of polymer nanoparticles can overcome the application limitations of NPs.

#### 3.2.3. Dendrimers

Dendrimers are synthetic polymers with a “tree-like” structure consisting of a small molecular core, internal cavities formed by multiple branches, and surfaces with plenty of functional groups. Compared to other polymers, dendritic polymers allow better control of their structure and hydrophilicity during formation, thus overcoming the limitations of low water solubility, permeability, and biocompatibility of drugs, while improving the in vivo circulation time and biodistribution [88]. Various dendrimers, such as polyamides (PAMAM), polyamide silicones, polypropylene imine, and sugar dendrimers, have been used in drug-release studies. Wang et al. [89] prepared an acetyl-modified PAMAM dendrimer (G5-Ac/Cur). The solubility of curcumin loaded with dendrimers was increased by nearly 200-fold, and it showed stronger toxicity and pro-apoptotic effects on A549 cells. Compared with other delivery strategies, the dendrimer is simple to prepare and can also improve the bioavailability of curcumin. In order to further achieve anti-tumor efficacy with high efficiency and low toxicity, the structure of the dendrimer can also be optimized. For example, Wang et al. [68] designed and prepared a GSH/Histone B dual-stimulated responsive dendritic polymer (HA-DTX-dendritic macromolecule, HADD) for DTX delivery. In this polymer, the glycodendron with a hyper-branched structure is connected to HA by a disulfide bond, which can grant HADD active targeting and GSH responsiveness specifically recognize tumor cells rich in CD44 receptors and stimulate the self-assembly morphology of HADD at high concentrations of GSH, promoting the tissue permeability of polymer nanoparticles to achieve accurate DTX delivery. Compared with free DTX, HADD demonstrated higher tumor growth inhibition (99.71%) in mouse models of MDA-MB-231 tumor vectors and had a good in vivo safety profile.

Although the use of dendritic macromolecule delivery strategies can improve the delivery efficiency of drug anti-tumor active ingredients, the conjugation of dendritic macromolecules to herbal active ingredients with active functional groups reduces the solubility of the entire molecule, and the non-degradability of dendritic macromolecules and the large cationic charge on the surface can lead to high cytotoxicity [90]. Therefore, only a few TCM active ingredients have been loaded into dendrimers, such as geranylgeranyl, genistein, and mycotoxins [91].

In conclusion, the polymer-based nano-delivery system has the characteristics of biodegradability, water solubility, biocompatibility, and easy modification, and is an ideal drug delivery material. In the future, more polymer nano-delivery systems can be developed to deliver drugs, proteins, and genetic materials to target tissues for cancer medicine or gene therapy.

#### 3.2.4. Biopolymer-Based Nanocarriers

Biopolymer-based nanocarriers are primarily composed of natural biopolymers derived from proteins and polysaccharides and their modifiers. These nanocarriers have good properties such as ease of modification, biorecognition, biodegradability, biosafety, and easy processing into gels [92,93]. In addition, the delivery system overcomes the limitations of traditional nanomaterials, i.e., the nanocarriers cannot be metabolically broken down in the body after drug release. It is worth noting that biopolymer-based nanocarriers with an optimized structure not only have the ability to target and control drug release but also the carrier can be naturally degraded in vivo and eventually excreted or participate in the metabolism of living organisms.

Thus, TCM active ingredients are increasingly being delivered to the patient via biopolymer-based nanocarriers. Common applications of biopolymer-based nanocoliters include:

(1) Chitosan, a natural biopolymer, has been developed for the encapsulated delivery of traditional Chinese medicine active ingredients such as curcumin [94] and trans-resveratrol [95] due to its better biodegradability and targeting. Xu et al. [96] designed a polymer chain with active targeting, pH response, and imaging capability to form multi-functional polymer CS-BT-HBS-CB micelles via self-assembly for in vivo delivery of paclitaxel. Structurally, HBS with an aggregation-induced emission (AIE) effect is helpful to monitor micellar carrier delivery. CLSM images show that the delivery system can be effectively internalized by MCF-7 cells, and the yellow fluorescence in the cells is gradually enhanced with the increase in incubation time. This may be the result of targeted delivery mediated by biotin (BT) in the polymer chain. In addition, the breakdown of the benzoate imide bond at weakly acidic pH triggers a responsive drug release, with 80.8% of PTX released in vitro at pH 5.0 and only 33.3% at pH 7.4. This responsive drug release property not only reduces the toxic side effects of PTX, but also increases the anti-tumor effect of the polymer delivery system, with a tumor inhibition rate of 66.9%. In general, this biopolymer-based delivery system designed based on chitosan has good biocompatibility and superior anti-tumor efficacy, showing broad application prospects.
(2) The hydrogels of biopolymers have a cross-linked polymer network that provides space for hydrophilic polymer chains to accommodate aqueous biofluids with good biocompatibility. Some of these hydrogels undergo phase changes when stimulated by the external environment; at the same time, the drug is released in a controlled manner with the change in its physical properties [97,98]. For example, curcumin can already be encapsulated in nanogels based on folic acid and casein for the treatment of skin cancer. Priya et al. [99] fabricated CUR-loaded nanogels (NGs) using a layer-by-layer technique (LbL) and modified NGs with folic acid (FA) and casein for drug delivery in skin cancer. Quantification of cell viability by MTT assay and light microscopy images showed that NG-scanned CURs were delivered directly to tumor cells, and the nanogels showed enhanced targeting ability to tumor cells and exhibited superior cytotoxicity in tumor cells due to folic acid receptor-mediated endocytosis, improving efficacy and reducing drug side effects.

### 3.3. Metallic Nanocarrier

Different inorganic nanocarriers have attracted increasing interest over the past few years due to their structural stability, biocompatibility, and non-toxicity in the biomedical field. Inorganic nanoparticles have shown great potential as carriers for traditional Chinese medicine antitumor active ingredients, particularly for slow-release drug applications.

For example, gold nanoparticles are characterized by easy surface modification and binding, which allows them to be loaded with drugs in non-covalent binding, and they also have good stability and biosafety [100]. For example, Ding et al. [101] took FeMOF NPs composed of TCPP (Fe) and zirconium clusters as the nanoplatform, and Au NPs formed a PEG-modified hybrid nano-delivery system by in situ generation anchored on the surface of MOF (PEG-Au/FeMOF@CPT NPs). The design of this delivery system is complex and novel. FeMOF NPs with a porous structure could effectively encapsulate the hydrophobic anti-tumor active ingredient CPT of Chinese medicine and increase the solubility and bioavailability of the drug. Interestingly, surface-anchored Au NPs not only improved the stability of nanocallers but also catalyzed intracellular glucose oxidation to produce hydrogen peroxide, provided energy for the Fenton reaction, effectively inhibited HepG_2_ cell growth, and realized the combined treatment strategy of CPT-mediated chemotherapy and hydrogen peroxide-mediated chemokinetic therapy (CDT). More importantly, due to the coordination of phosphates and zirconium in the cell, these particles exposed to the decomposition of high concentrations of phosphates in TME can rapidly release CPT, with a cumulative release of more than 80%. In vivo antitumor studies in HepG_2_ tumor-bearing nude mice showed that NPs had satisfactory therapeutic properties, the tumor inhibition rate was 85.6%, and H&E and TUNEL staining exhibited the highest level of apoptosis and necrotic cells in tumor tissues, showing good application potential in cancer therapy. In brief, AuNPs, as drug carriers, are widely used in drug delivery because of their good biological safety, strong stability, and easy surface functionalization. In addition, Au NPs can be accumulated in tumors driven by magnetic force to realize the integration of diagnosis and treatment of cancer, and the FDA has approved gold nanoparticles to enter clinical research [102,103].

In addition to gold nanoparticles, mesoporous silica nanoparticles (MSNs) are also common inorganic nanocarriers. Due to the ordered pore structure and adjustable specific surface area of mesoporous silica nanoparticles, it is possible to load large amounts of drugs into mesoporous silica nanoparticles. Moreover, the excellent biocompatibility, stability, and easy modification of the nanocarriers mean MSNs are widely used in the delivery of antitumor components of TCM in vivo [104,105]. In addition, MSNs can also be mixed with other types of nanocarriers to form a two-carrier delivery system to achieve a dual-drug strategy. For example, Manjusha et al. [106] prepared a novel MSN-gated PF127/PP123 mixed micelle based on two carriers: Mesoporous silica nanoparticles (AMSN) and micelles formed by the self-assembly of two block copolymers. DOX could be encapsulated in the pores by the AMSN with a porous structure through electrostatic attraction (EE = 92.12%) at pH 7.4, and PTX was loaded inside the micelle and exhibited high load capacity (EE = 95%). Notably, the RGD peptide-modified MM-PTX micelle acted as a gated AMSN to actively recognize the highly expressed integrin αvβ3 in breast cancer cells, reducing the toxicity of the antitumor component to normal tissues. Both in vitro cytotoxicity and cell death staining experiments proved that the mixed delivery system had good tumor inhibition ability with concentration dependence, which may be the result of the synergistic antitumor effect of the two drugs.

Despite the advantages of MSNs, such as good biocompatibility, porousness, increased specific surface area, and easy modification, drug encapsulation inside MSNs is prone to drug leakage and limited drug loading volume [107]. To overcome the limitations of MSNs in drug delivery, the development of hollow mesoporous silica nanoparticles (HMSNs) can effectively improve the loading capacity of drugs, and the bilayer structure of these nanocarriers can realize the co-loading of hydrophilic and hydrophobic drugs. For example, Yin et al. designed HMSNs with both active targeting and multiple response capabilities for the codelivery of PTX and 5-fluorouracil (5-FU). The system was able to recognize the folate receptor overexpressed on the surface of breast cancer for precise drug delivery. When effectively internalized by MCF-7 cells, HMSNs triggered the responsive release of the dual drug, enabling the synergistic therapeutic effect of the two drugs, which was an effective strategy for two-drug delivery [108]. In addition, in order to solve the problem of drug leakage, the carrier structure can be modified by designing MSNs with membrane encapsulation [109] or plugging the pores in the carrier [110]. Li et al. prepared ZnO quantum dot-sealed HMSNs for the co-delivery of CPT and DOX. Structurally, the aminated ZnO was connected to -COOH on the surface of the nanoparticles via amide bonds to firmly seal the nanoparticles and prevent leakage during drug delivery. TEM images demonstrated that the HMSNs were successfully sealed [111].

Magnetic nanoparticles constitute a kind of nanocarrier formed by magnetic materials, which has the characteristics of good biocompatibility and magnetic conductivity. For example, magnetic Fe_3_O_4_ nanoparticles are widely used in drug delivery because of their good chemical stability and low toxicity. Wang et al. developed a magnetic Fe_3_O_4_ nanoparticle loaded with luteic acid (GA). This nanoparticle had a spherical structure of 20 nm, which could effectively avoid the clearance of the endothelial reticular system and the metabolic effect of the kidney. More importantly, GA could act as an inhibitor of the E26 transformation-specific sequence-1 (ETS1) transcription factor, and Western blot results showed that the expression level of ETS1 and the levels of cyclin D1, u-PA, and VEGF in Panc-1 cells treated with GA-MNP-Fe_3_O_4_ groups were significantly downregulated. It was further demonstrated that the nano-delivery system could inhibit the proliferation and migration of pancreatic cancer cells mediated by the ETS1 transcription factor [112].

Nanotubes with strong cellular uptake, high permeability and retention effects in solid tumors, and easy binding to chemotherapeutic drugs can be loaded with various antitumor active ingredients of TCM, such as PTX and resveratrol [113,114]. Koh et al. [115] designed targeted-delivery functionalized carbon nanotubes (fCNTs)-encapsulated topoisomerase I inhibitor camptothecin (CPT). To target CPT delivery to AVB3-expressing tumor cells, the cycloarginine aspartate (RGD) peptide was covalently coupled to the CNT surface. The results showed that CPT@fCNT-RGD could be successfully delivered to A375 cells. Compared with the non-targeted CPT@fCNT, the anticancer effects of actively targeted carbon nanotubes in 2D and 3D cultures were 3.78-fold and 3.02-fold, respectively.

**Figure 3:**
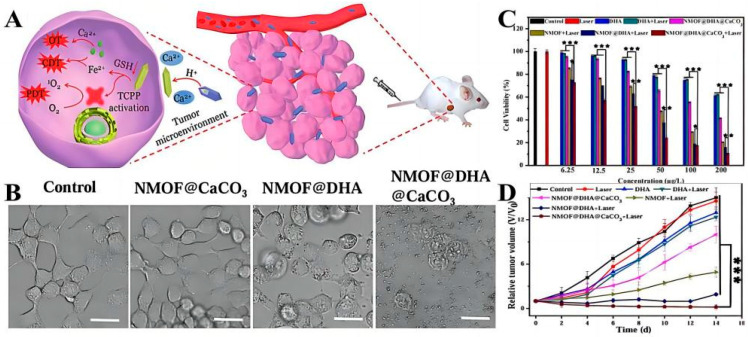
(**A**) Model diagram of programmed drug release. (**B**) Morphology of 4T1 cells with different treatments. The scale bar is 25 μm. (**C**) MTT assay results of mice breast cancer cells treated with different protocols. (**D**) Tumor growth curves of mice after using different treatment regimens. (* *p* < 0.05, ** *p* < 0.01, *** *p* < 0.001). Adapted with permission from Ref. [116]. Copyright 2019, Wiley-VCH.

Metal-Organic Frames (MOFs) are widely used for drug loading delivery due to their extremely high specific surface area, adjustable pore size, and surface properties. Among them, iron-based MOFs have promising applications in the development of iron-involved multimodal tumor treatment strategies due to the combination of the advantages of MOFs and the role of iron in tumor therapy. For example, Wan et al. developed an NMOF structural delivery system for CaCO_3_ mineralization based on iron-based MOF to realize the programmed release of Dihydroartemisinin (NMOF@DHA@CaCO_3_) [116]. When the system reached the tumor site, the redox reaction between GSH and Fe^3+^+triggered the release of DHA and the activation of TCPP, thus realizing Fe^2+^-DHA-mediated chemical kinetic therapy (CDT) and TCPP-mediated PDT (Figure 3A). The experimental in vitro results showed that untreated cells and cells treated with NMOF@CaCO_3_ were in a normal state, while cells treated with NMOF@DHA dissolved and died (Figure 3B). Cytotoxicity results also proved that these nanoplatforms could significantly enhance the antitumor effect in a dose-dependent manner (Figure 3C). Finally, the antitumor efficiency of NMOF@DHA@CaCO_3_ in vivo was studied with 4T1 tumor-bearing mice as an animal model. The results showed that the delivery system had good biocompatibility and an enhanced antitumor effect (Figure 3D), and the tumor did not recur and metastasize in a later stage of treatment and could even be completely eliminated, which provided a reference for the design of a redox-responsive drug-delivery system in vivo in the future.

Graphene also has potential clinical applications in the delivery of active antitumor components in Chinese medicine. To improve quercetin’s antitumor specificity and efficacy, Zhang et al. [117] delivered quercetin loaded with polyether amine and HA-modified graphene oxide (GO). The modified GO was pH-sensitive and remained biocompatible at GO concentrations of up to 350 μg/mL. The delivery system’s antitumor effect and longer-lasting efficacy were double compared to free leupeptin.

### 3.4. Miscellaneous Approaches

**Figure 4:**
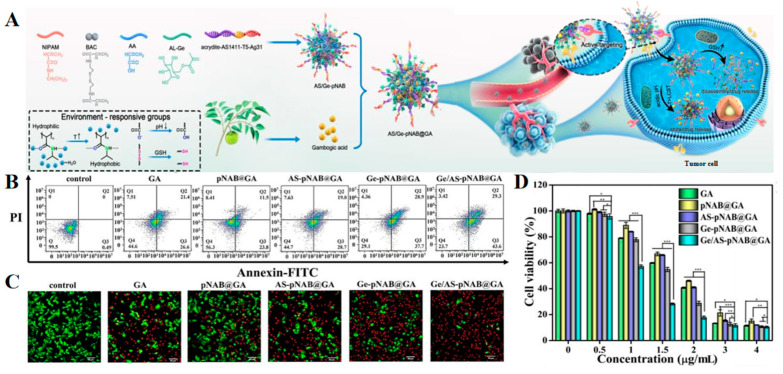
(**A**) Schematic overview of the preparation and application of multifunctional microgels for precisely targeted delivery of GA. (**B**) The apoptosis of HepG_2_ cells induced by different groups was analyzed by Annexin V-FITC/PI staining. (**C**) Confocal laser scanning microscope (CLSM) images of live/dead cell staining assay in different groups. (**D**) The cytotoxicity of HepG2 cells exposed to various concentrations of different groups (GA dosage: 0, 0.5, 1, 1.5, 2, 3, and 4 μg/mL) for 24 h. Data are presented as mean ± SD (*n* = 3). * *p* < 0.05, ** *p* < 0.01, *** *p* < 0.001. Adapted with permission from Ref. [122]. Copyright 2023, American Chemical Society.

In addition to the traditional delivery systems, nano-delivery systems for antitumor active ingredients in Chinese medicine also include microgels, nanocapsules, liquid crystals, and nanofibers. Microgels are a kind of highly cross-linked colloidal particles with sizes ranging from tens of nanometers (also called “nanogels”) to micrometers, which are produced by polymerization or the cross-linking reaction of polymer materials [118,119]. Microgels have the characteristics of high loading capacity, large volume deformation, and controllable size, and have been widely used in drug delivery in recent years [120,121]. However, the preparation process of most microgels is complicated, and in general, nanocarriers will gradually release the loaded drugs during long-term circulation in vivo, resulting in reduced drug efficacy. In order to further improve the delivery efficiency, our research group used a simple free radical precipitation polymerization method to construct a DNA microgel-silver nanocluster system (AS/Ge-pNAB microgels) with dual targeting, multiple environmental (pH, glutathione, and temperature) responsiveness, and real-time monitoring capability, and realized accurate, controllable, and targeted delivery of the anti-tumor drug GA with high efficiency (Figure 4A) [122]. After tail vein injection, microgels could specifically recognize the over-expressed nucleolus and asialoglycoprotein receptor on the surface of hepatocellular carcinoma cells through the dual targeting effects of surface-modified aptamer and galactose and deliver GA to the tumor site accurately. The responsive release of GA could be triggered by weak acid pH and a high concentration of GSH in the tumor microenvironment, thus killing tumor cells. Compared with free GA and single-targeted microgel group, the results of cytotoxicity and apoptosis exhibited that the dual-targeted DNA microgel had a stronger therapeutic effect, and the apoptosis rate reached 72.9% (Figure 4B,D). The living and dying staining experiment of cells also showed that the red fluorescence after AS/Ge-pNAB@GA treatment was significantly higher than that of other groups, proving the excellent therapeutic effect of the microgel system on tumors (Figure 4C). In addition, the dual targeting system also had good in vivo safety and strong tumor inhibition ability, and the tumor volume on day 17 was only 37 mm^3^. These results demonstrated that the multifunctional microgel had potential advantages as a GA delivery system for cancer treatment. In the future, the microgel system can also be used to package other active ingredients of traditional Chinese medicine for the treatment of a series of other cancers.

Nanocapsules are colloidal dispersion systems made of natural or synthetic polymeric materials as the outer shell and active substances as the inner core, which have a core–shell structure and can be fabricated into sealed microspheres via processing. Researchers have recently developed multifunctional nanocapsules or modified ligands in a variety of ways in order to achieve better antitumor results. Debasree Ghosh et al. [123] used poly(lactic acid)-ethyl cross-ester copolymer nanomicrocapsules loaded with CUR and evaluated the inhibition of diethylnitrosamine (DEN)-induced hepatocellular carcinoma (HCC) in rats. Compared to free CUR, the nanomicroencapsulated CUR significantly counteracted oxidative damage and produced significant inhibition of rat hepatocellular carcinoma cells as a potential oral agent. Electrospun fibers can also be used as carriers to deliver drugs, and Luo et al. [124] combined acetal groups with biodegradable poly(D, L-propyl cross-ester)-poly(ethylene glycol) to form an acid-intolerant polymer to load hydroxy camptothecin into electrospun fibers for intra-tumor therapy. The fibers were implanted into tumors and were able to show rapid targeting of tumors, producing a greater killing effect on tumor cells and significantly improving the survival time of animals, so the acid-intolerant electrospun fibers are expected to be excellent implants for local treatment of inoperable and recalcitrant tumors. So far, there has been little research on these nano-delivery systems, but they have good prospects for development.

In conclusion, the wide variety of inorganic nanocarriers can overcome the instability problems of lipid-based nanocarriers, such as easy oxidation and hydrolysis. In addition, more properties of inorganic nanocarriers can be used for various types of antitumor therapy, such as photodynamic therapy, photothermal therapy, etc., which is a promising strategy for drug delivery.

## 4. Covalently Combined Pro-Drug-Delivery Systems

A pro-drug refers to a compound that is obtained by modifying the chemical structure of drugs, which remains inactive during transport and its activity is restored by certain conditional stimulation in vivo to exert its efficacy [125,126]. Compared with normal tissues, TME has the characteristics of weak acidity, hypoxia, high GSH concentration, etc. [127]. According to these characteristics, a pro-drug nano-delivery system with a tumor microenvironment response can be designed to realize the controlled drug release by endogenous stimulation after connecting the delivery carrier and the drug through covalent bonds and achieve targeted “on-demand administration” in the diseased area of the body, avoiding drug leakage, greatly increasing drug accumulation in tumor cells, and reducing systemic toxicity [128].

### 4.1. pH-Responsive Pro-Drug-Delivery Systems

The pH-responsive pro-drug-delivery system is a nano-delivery system, which includes pH-responsive micelles, pH-responsive nanoliposomes, and pH-responsive gels. These nanoplatforms can passively or actively target tumors and realize pH-sensitive drug release, thus significantly improving the curative effect and reducing toxic and side effects [129,130]. Du et al. [131] synthesized a pH/tissue protease B-responsive PTX pro-drug nano-delivery system (HRNs) with a stable nanostructure (40 nm). HRNs could aggregate at the tumor site via passive targeting mediated by the EPR effect and then rapidly dissociate into macromolecular adducts (5 nm) in a weakly acidic environment, facilitating deep tumor diffusion and systematically overcoming barriers in the delivery process to precisely deliver PTX to tumor cells. The co-localization of HRNs and lysosomes in 4T1 cells was observed using laser confocal microscopy. The results confirmed that HRNs were efficiently delivered intracellularly to the lysosomes, which were then degraded and released PTX into the cytoplasm. The results of animal experiments showed that HRNs have powerful therapeutic effects in 4T1 and B16OVA tumor models.

Although the EPR effect can promote the targeted delivery of antitumor active ingredients of TCM, it is complex and shows significant individual differences among patients. Therefore, the strategy of targeting tumor tissue via only the EPR effect is still insufficient in clinical applications, and it is essential to develop a more effective method to deliver TCM. Of note, the tumor microenvironment can produce the expression of specific components or the over-expression of some components, and according to this feature, the targeting ligands recognizing specific receptors in tumor sites can be modified on the surface of the nano-delivery system. The recognition of unique receptors and corresponding ligands on target cells provides a molecular basis for the “recognition and binding” function of nanocarriers, which not only enhances the targeting and internalization ability of nanocarriers to tumor cells but also improves the in vivo circulation time, tissue distribution, and tumor cell uptake of the nano-delivery system [132,133]. For example, Li et al. [134] constructed a pH-responsive pro-drug (Ha-Co-O-PPT) system with active tumor targeting by esterifying podophyllotoxin (PPT) with hyaluronic acid (HA). The pro-drug spontaneously assembled into nanoscale spherical micelles (HP) in an aqueous solution and showed good serum stability and blood compatibility. HA could bind to HA receptors overexpressed by tumor cells to produce the active targeting effect, thus improving the uptake efficiency of tumor cells to micelles with an efficiency of more than 97%. In the weakly pH tumor environment, the micelles released ester bond-breaking PPT in large quantities, releasing cytochrome C from mitochondria into the cytoplasm, further activating the caspase cascade and inducing apoptosis. Tumor suppression was up to 85% with negligible systemic toxicity.

**Figure 5:**
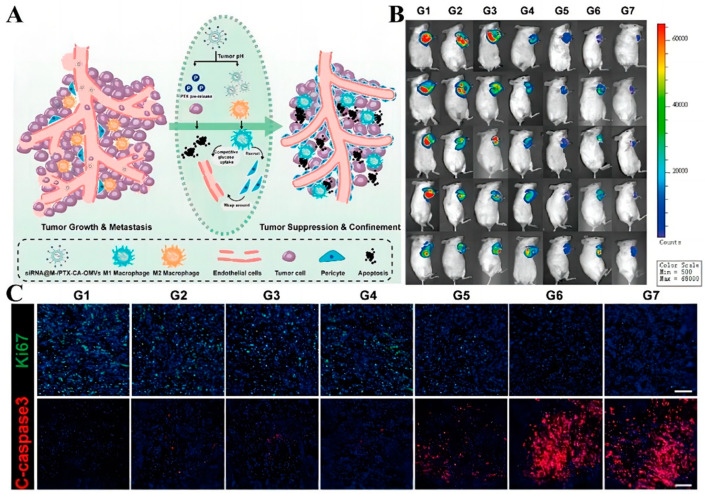
(**A**) Diagram of the mechanism of action of siRNA@M-/PTX-CA-OMV. (**B**) In vivo imaging of 4T1 tumor xenografts mice 14 days after administration. (**C**) Histological images of 4T1 tumor xenografts. Caspase 3: Apoptotic tumor cells. Ki67: Growing tumor cells (scale bar = 100 μm). Adapted with permission from Ref. [136]. Copyright 2021, American Chemical Society.

In addition, modulation of the tumor microenvironment can also overcome barriers to drug delivery. For example, metabolic interactions between different types of cells in the tumor microenvironment can promote tumor growth and competitively suppress anti-tumor immunity [135]. Therefore, modulating the metabolic microenvironment of tumors is also a practical approach. Due to tumor heterogeneity, there is an urgent need to develop a drug-delivery system that targets different cellin microenvironments separately to achieve synergistic therapeutic effects. Therefore, Guo et al. [136] produced a drug delivery platform (M-/CA-OMV) using bacterial outer-membrane vesicles (OMVs) to co-deliver the pH-sensitive PTX pro-drugs and siRNAs that activate DNA damage response 1, sequentially targeting various cells in the TME to achieve the combined application of chemotherapy and gene therapy (Figure 5A). Structurally, the pH-sensitive linker cis-aconitic anhydride was used to link PTX and DSPE-PEG for responsive drug release, reducing excessive drug uptake by macrophages and minimizing toxic side effects. A portion of PTX was released in TME with weakly acidic pH to kill tumor cells by chemotherapeutic drugs, thus blocking the metabolic stress induced by tumor cells. The remaining PTX is phagocytosed by M2 macrophages used to increase glycolysis levels and thus inhibit tumor metastasis. This reduces the unnecessary uptake of PTX by macrophages. To evaluate the in vivo anti-tumor effect of this system, an in situ model of TNBC in female mice was established to observe the tumor growth after drug administration. As shown in Figure 5B, the siRNA@M-/PTX-CA-OMVs groups had good in vivo antitumor ability with minimal tumor volume. In addition, caspase3 signaling increased after treatment with this system, showing significant red fluorescence compared with other treatment groups, while ki67 had less green fluorescence, indicating that the system had a satisfactory antitumor proliferation effect (Figure 5C).

### 4.2. Reduction-Responsive Pro-Drug-Delivery Systems

The concentration of reduced glutathione (GSH) in the tumor microenvironment can reach 2–10 mM, much higher than the 2–10 μM in normal tissues. Since the GSH concentration in tumor tissues is much higher than in normal tissues, the large difference in the GSH concentration facilitates the application of reduction-responsive pro-drug-delivery systems [137]. Disulfide bonds are the most common reduction-reactive chemical bonds connecting nanocarriers and antitumor drugs and can be broken reductively in the tumor microenvironment with high GSH concentrations, thus controlling drug release. This delivery system is highly targeted, has excellent drug loading capacity, significantly prolongs the cycle time of PTX, improves bioavailability, and reduces the systemic toxicity caused by chemotherapeutic agents. Yi et al. [138] synthesized GSH-sensitive dimeric paclitaxel (diPTX) pro-drugs and obtained novel composite nanoparticles (diPTX@Fe&TA) by depositing Fe&TA network complexes that had great PTX loading capacity. When it was internalized by tumor cells, nanoparticles could be continuously exposed to an acidic lysosomal environment and high levels of GSH, allowing the successive release of diPTX and PTX. In contrast, PTX is hardly released under physiological conditions. In vivo experiments have demonstrated its high stability, high drug load, and high targeting characteristics, allowing it to effectively inhibit tumor growth in vivo with minimal organ toxicity. Li et al. [139] designed and synthesized a disulfide-bridged DHA dimer precursor (DHA_2_-SS) that self-assembles into nanoparticles (SS NPs). These nanoparticles had a high DHA content (>90%) and exhibited a sensitive redox response, allowing the rapid release of DHA in TME, enhanced tumor accumulation, and effective endocytosis. In vitro tumor experiments showed the significant modulation of intracellular carbohydrate, amino acid, and lipid metabolism after treatment with SS NPs, suggesting that SS NPs may achieve antitumor effects through the mitochondrial apoptotic pathway and induction of metabolic reprogramming in tumor cells. Animal experiments showed that SS NPs had more pronounced cytotoxicity and better antitumor effects compared with the control group.

**Figure 6:**
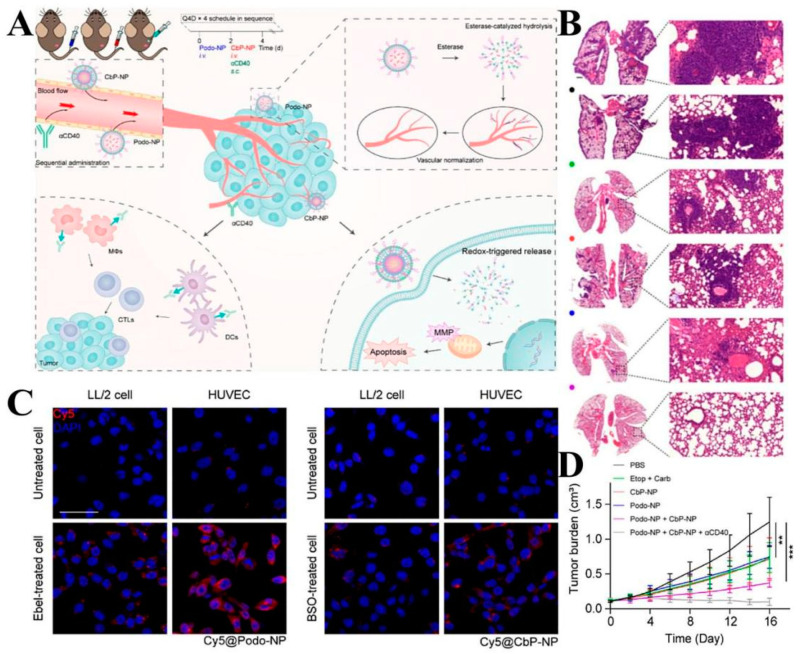
(**A**) Diagram of the therapeutic mechanism of nanoparticles (**B**) H&E-stained sections of lungs. (Scale bars: left: = 100 μm; right: = 50 μm). (**C**) Effect of esterase and GSH depletion on the degree of intracellular dissociation of both delivery systems (Scale bar: 50 μm). (**D**) Changes in tumor volume in mice after treatment using different protocols. ** *p* < 0.01, *** *p* < 0.001. Adapted with permission from Ref. [141]. Copyright 2020, American Chemical Society.

This delivery system can also be loaded with multiple or individual drugs to disrupt tumor survival conditions, activate immune responses, and better inhibit tumor cell growth and metastasis [140]. Ling et al. [141] designed two delivery platforms (Podo-NP and CBP-NP) for the delivery of the esterase-sensitive podophyllotoxin pro-drug (Podo) and the redox-sensitive carboplatin pro-drug (Carb). Podo-NP was decomposed and released under the stimulation of a high esterase concentration in TME to inhibit tumor angiogenesis and enhance the immune response by promoting vascular remodeling, while CBP-NP was internalized by tumor cells and released Carb to kill tumor cells by blocking cell proliferation and division. The CD agonist was used to process the relevant antigens released by dead tumor cells and thereby reverse the immunosuppressive microenvironment (Figure 6A). To verify the responsiveness of the two nanoplatforms, HUVEC was incubated with esterase inhibitors and γ-glutamyl transpeptidase GSH synthesis inhibitors, respectively, and intracellular fluorescence intensity was observed. The results demonstrated that once taken up by the cells, Podo-NP and CbP-NP were triggered by esterases and reductants to release their payloads inside the cells (Figure 6C). In vivo experiments also showed that this combined therapeutic system was able to significantly inhibit tumor growth (Figure 6D), and in vitro H&E staining showed reduced nuclear division of tumor cells in the Podo-NP + CbP-NP + αCD40 group (Figure 6B). Zhang et al. [142] designed a polyethylene glycol lipid self-assembled nanoparticle (S-NP-CPT) for loading GSH-sensitive CPT pro-drugs. The nanoparticles were stimulated by high concentrations of GSH from TME to release CPT, and in vitro experiments showed that the cytotoxicity of S-NP-CPT was nearly twice that of the control group, which may be related to its activation of the STING pathway. In in vivo experiments, S-NP-CPT increased the drug’s blood circulation time by 80-fold with lower systemic toxicity. Meanwhile, the tumor suppression rate of S-NP-CPT was twice as high as that of free CPT. By analyzing animal models, it was hypothesized that S-NP-CPT could significantly promote dendritic cell maturation and CD cell infiltration, which resulted in its superior tumor suppressive effect.

Apart from that, this drug-delivery system can work synergistically with other treatments to better inhibit tumor growth. Hao et al. [143] constructed a drug delivery platform (PTX-SS-HPPH/Pt@RGD-NP) by bridging PTX with a photosensitizer using disulfide bonds to prepare a GSH-responsive pro-drug and then modified it with DSPE-PEG and RGD peptide while using PtNP to overcome the therapeutic limitations of bladder cancer due to hypoxia. The results showed that the platform could target the tumor by RGD and release the drug in TEM, and then generate a large amount of ROS under 660 nm laser irradiation while significantly enhancing PDT efficacy in the presence of PtNP, achieving a synergistic effect of chemotherapy and PDT for potent tumor killing.

### 4.3. Hypoxia Responsive Pro-Drug-Delivery Systems

**Figure 7:**
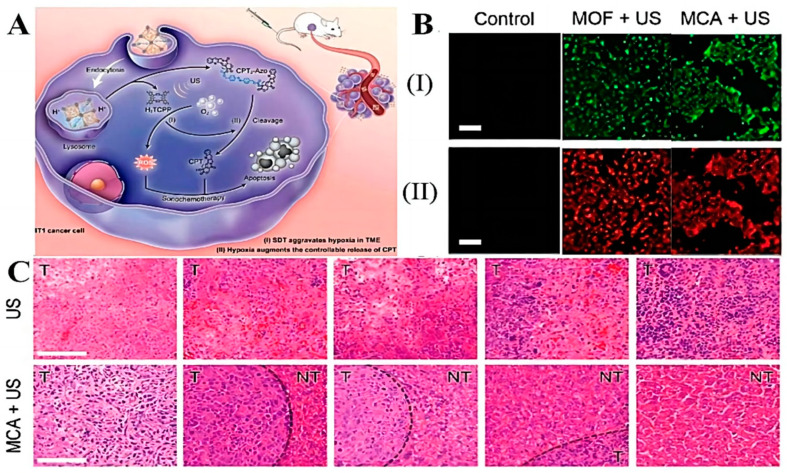
(**A**) Illustration of MCA treatment mechanism. (**B**) Fluorescence images of 4T1 cancer cells incubated with MOF NPs or MCA nanoamplifier under US actuation and then stained with the ROS/hypoxia detection probe: (**I**) ROS (**II**) Hypoxia level Scale (bar: 200 µm). (**C**) H&E staining of liver tissue on days 0, 4, 8, 12, and 16. (T: Tumor NT: Non-tumor). Adapted with permission from Ref. [147]. Copyright 2022, American Chemical Society.

The overproliferation of tumor cells leads to increased oxygen consumption, while the lack of oxygen supply causes generalized tumor hypoxia. [144]. Based on this feature, a hypoxia-responsive drug-delivery system can be designed. Hypoxia-responsive nanoparticles can reduce the effect of the tumor-hypoxic environment on PDT therapy, while PDT-induced hypoxia exacerbation can enhance the therapeutic effect of hypoxia-activated pro-drugs. Zhou et al. [145] prepared a novel nano-delivery system (Ce6/PTX_2_-Azo NP) by encapsulating a hypoxia-activated PTX pro-drug (PTX_2_-Azo) in a peptide copolymer modified with the photosensitizer Ce6, which, on the one hand, prevented premature drug leakage in vivo, and on the other hand, had hypoxia sensitivity under hypoxic conditions. Bioreductive pro-drugs with hypoxia-sensitive molecules can be activated for release. As photodynamic therapy consumes oxygen to generate reactive oxygen species, it exacerbated the hypoxic condition of tumor cells. Further, it promotes the release of antitumor drugs with significant antitumor activity. In vivo, animal experiments showed that the Ce6/PTX_2_-Azo NP+L group showed the strongest tumor inhibition and the smallest tumor volume, with the complete disappearance of tumors in mice. The synergistic effect of PDT and chemotherapy with an enhanced antitumor effect was further confirmed by the mean isolated tumor weight and apoptosis of tumor cells observed by HE staining. Yang et al. [146] fabricated a multifunctional molecular pro-drug BAC nanoparticle (BAC NPs) by linking camptothecin (CPT) with diphenyl thiophene (BODIPY) photosensitizer through an azobenzene chain and encapsulating it in monomethoxy polyethylene glycol-b-polycaprolactone (mPEG-b-PCL). Compared to CPT, BACs enabled higher colloidal stability and can better reduce unwanted drug leakage. Internalized by tumor cells, bAC NPs could act synergistically with PDT to destroy nearby tumor cells and cause acute hypoxia, allowing the nanoparticles to cleave azobenzene junctions stimulated by azoreductase, which in turn accelerates the release of CPT. The released CPT penetrated deeply into tumor cells and greatly improves the limitation of tumor killing by PDT due to hypoxia. In vivo, experiments showed that BAC NPs synergized with PDT to produce significant antitumor effects. Zhuang et al. [147] obtained a hypoxia-sensitive drug delivery platform (MCA) that can synergize with sonodynamic therapy (SDT) by loading azo-linked CPT pro-drugs (CPT_2_-Azo) into MOF NPs filled with acoustic sensitizers. Under the influence of ultrasound, intracellular oxygen was converted to ROS to promote SDT treatment, which in turn further deepened cellular hypoxia to promote Azo bond breakage for more CPT release deeper into the tumor (Figure 7A). The CLSM observed the degree of ROS production and hypoxia aggravation in 4T1 cells and found that MCA could effectively produce ROS under US driving, showing green fluorescence. Due to ROS production accelerated hypoxia in tumor cells, the red fluorescence in cells was enhanced (Figure 7B). This was also demonstrated in animal experiments, where CPT_2_-Azo resulted in the most tumor-killing and inhibitory effects in hepatocellular carcinoma compared to other groups (Figure 7C), and since CPT_2_-Azo was not converted to the more toxic CPT in normal cells, MCA produced little non-essential toxicity.

### 4.4. Reactive Oxygen Responsive Pro-Drug-Delivery Systems

Reactive oxygen species (ROS) is a general term for oxygen molecular derivatives, including superoxide (O^2−^), hydroxyl (-OH), singlet oxygen (^1^O_2_^−^), and hydrogen peroxide (H_2_O_2_). In normal cells, physiological levels of ROS are important components for maintaining vital activities and cellular communication, while the redox state in tumor areas is significantly higher than that in normal tissues, and high concentrations of ROS can cause cellular damage. Based on this principle, an ROS-responsive nanoprecursor drug-delivery platform can be developed for tumor therapy. The thioketone bond (TK) is a common ROS chemical bond, but the low concentration of ROS inherent in the body cannot cleave this bond. Photosensitizers are effective in generating ROS that greatly increase the level of reactive oxygen species, thereby inducing TK bond breakage. Therefore, a delivery system based on an ROS-sensitive photosensitizer and the TK bond can be designed for responsive drug release by local irradiation of a tumor. Hao et al. [148] created an ROS-responsive pro-drug by linking CPT to HPPH through the TK bond and loading it with PtNP. When the pro-drug entered the tumor, a large amount of ROS was generated by using 660 nm laser irradiation, which broke the TK bond and promoted the release of CPT deep into the tumor. Meanwhile, H_2_O_2_ decomposition by PtNP to produce oxygen was accelerated, which relieved tumor hypoxia and improved PDT efficiency. Both in vivo and in vitro experimental results showed that this therapeutic strategy significantly improved the targeting and in vivo circulation time of CPT, and effectively inhibited the proliferation of colon cancer.

**Figure 8:**
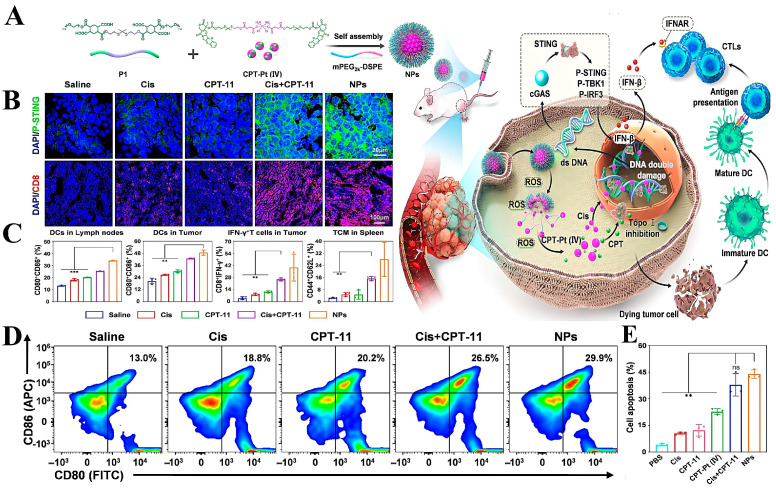
(**A**) Diagram of the mechanism of immune activation by NPs. (**B**,**C**) Expression of p-STING and infiltration of immune cells in tumors; histograms of DC cell and interferon distribution in different protocols (** *p* < 0.01, *** *p* < 0.001). (**D**) CLSM plots of tumor sections from mice. (**E**) auantification of apoptosis via FCM on CT26 cells treated with Cis, CPT-Pt (IV), Cis + CPT-11 and NPs (10 μM Pt, 20 μM CPT-11) at 24 h. Adapted with permission from Ref. [149]. Copyright 2022, the authors.

In addition, the ROS responsive delivery system can be combined with tumor immunotherapy to activate the immune response and provide deep killing of tumor cells. Cao et al. [149] designed ROS-responsive nanoparticles (NPs) based on ROS-sensitive polymers (P1) for the encapsulation of a novel pro-drug (CPT-Pt(IV)). The NPs could target the release of CPT and cisplatin at high concentrations of ROS in TME, which caused dual damage to the tumor DNA and thus promoted the opening of cGAS-STING channels (Figure 8A). Subsequent in vitro experiments and metabolomic analysis showed that CPT-Pt(IV) promoted the expression of pathway-associated proteins and activation of CD cells, thereby promoting tumor immunity (Figure 8B,C). Compared to controls, rectal cancer mice injected with NPs showed little systemic toxicity and obtained the greatest reduction in tumor volume.

### 4.5. Multi-Response Pro-Drug-Delivery Systems

Since malignancy is a complex disease, strategies combining multidimensional therapeutic modalities have obvious advantages so that two or more stimulus-responsive units can be introduced in a single pro-drug-delivery system to maximize the drug’s therapeutic effect. Li et al. [150] developed a transferrin receptor-targeted redox/pH dual-responsive allantoid podophyllotoxin (PPT). Pre-PPT covalently coupled T7 peptide-modified PEG or methoxy polyethylene glycol (mPEG) via disulfide bonds and mixed well to obtain uniformly dispersed and stable hybrid micelles (PEG-SS-NPs). In vitro, cytotoxicity assays showed that the resistance index of PEG-SS-NPs against different drug-resistant tumor cell lines was 270-fold lower than that of PTX or polyene PTX. Compared to controls, PEG-SS-NPs significantly improved cellular uptake and increased the maximum tolerated dose. In vivo, it was shown that PEG-SS-NPs significantly enhanced the antitumor effect against xenogeneic tumors. Wang et al. [151] synthesized GSH-responsive and pH-responsive cisplatin pro-drug and PTX co-loaded nanoparticles (DDP-P/PTX NPs). The uptake of DDP-P/PTX NPs and DDP/PTX NPs by A549/DDP cells was more than 70%. This result was found at pH 5.0 and could release more drugs than at pH 7.4, and the release rate was further accelerated after increasing the GSH concentration. In vitro experiments showed that DDP-P/PTX NP showed significant cytotoxicity and higher cell growth inhibition than DDP/PTX NP. The in vivo drug distribution of nanoparticles was higher in tumor tissues and lower in the heart and kidney compared to controls, making it promising as an ideal drug for treating non-small cell lung cancer. Yin et al. [152] developed a GSH/ROS dual-sensitive pro-drug (GR-BCP) for targeted drug release and enhanced antitumor efficacy, with a side chain consisting of PEG and CPT.

**Figure 9:**
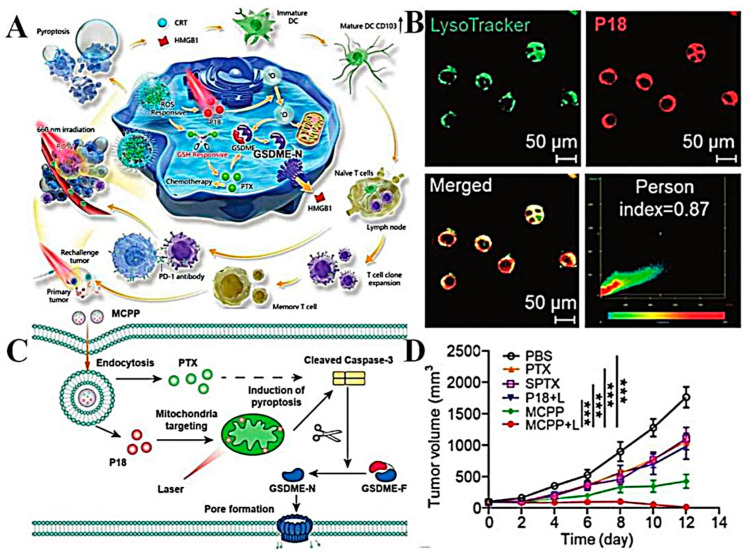
(**A**) Diagrammatic representation of the role of MCPP in tumor cells. (**B**) Co-localization of MCPP and lysosomes in CT 26 cells. (**C**) Schematic illustration of pyroptosis induced by MCPP+L. (**D**) Tumor growth curves for each group (*n* = 6). *** *p* < 0.001. Adapted with permission from Ref. [154]. Copyright 2021, the authors.

A GSH or ROS nonreactive pro-drug system was also prepared for comparison. The pro-drug systems with dual GSH and ROS response were able to target tumors better than the post-drug systems without a GSH or ROS response and allow for controlled drug release. The single-response pro-drug system and GR-BCP both showed similar antitumor effects in vivo. However, GR-BCP produced the best tumor suppression with the fewest side effects. Wang et al. [153] synthesized pH-responsive pro-drugs (PEG_2K_-NH-N-DOX), GSH-responsive pro-drugs (PEG_2K_-S-S-CPT), folate receptor-targeting polymers (FA-PEG_2K_-L8 and FA-PEG_2K_-TOS), and T1-enhanced MRI contrast agents (Gd-DTPA-N16-16) and wrapped them in CombrestatinA 4 (CA 4) to obtain multifunctional nanoparticles (FTDCAG NPs), which can control the release in time and space. FTDCAG NPs accumulate in tumor tissues through EPR effects, bind specifically to cells overexpressing the folate receptor (FA) to release CA4 to specifically disrupt angiogenesis, then release CA4 through receptor-mediated endocytosis into tumor cells, degraded in a high-GSH and low-pH environment, and rapidly release DOX and CPT for synergistic multidrug therapy. In addition, they found that the relaxation time of FTDCAG NPs was 3.86-fold longer than that of clinical Gd-DTPA, with brighter T1-weighted imaging in vitro and in vivo, which could be used for MRI tracking of nanoparticles to locate tumors and quantify the accumulation of NPs. Xiao et al. [154] used ROS-sensitive MPEG, wrapped with a glutathione-responsive paclitaxel pro-drug (SPTX) and the photosensitizer purpurin 18 (P18) and self-assembled to form nanoparticles to form a dual ROS/GSH-responsive system (MCPP) (Figure 9A). MCPP entered tumor cells via the endocytic pathway, with the nano pro-drug escaping from lysosomes and targeting mitochondria, which was consistent with the CLSM image (Figure 9B). The cytotoxic ROS-induced apoptosis produced by the photoactivation of the photosensitizer makes MCPP significantly more toxic. In addition, MCPP systems induced tumor cell thermalization via chemotherapy-photodynamic therapy. The heated tumor cells released damage-associated molecular patterns (DAMPs), initiated adaptive immunity, increased the efficiency of immune checkpoint blockade, achieved tumor regression, generated immune memory, and prevented tumor recurrence. In vivo anti-tumor research proves that MCPP+L has the most effective tumor control ability, and the tumors of mice treated with MCPP+L almost disappeared after 10 days (Figure 9D), and the weight of each treatment group showed no significant change, which shows good safety. In addition, the anti-tumor mechanism of this system was studied and it was found that chemical photodynamic therapy and the controlled release of PTX synergistically induced gas protein E (GSDME)-related scorch death. Caspase-3 was able o activate GSDME, and the N-terminal fragment of GSDME formed holes in the plasma membrane, which led to cell death and the release of DAMPs (Figure 9C). The released DAMPs could promote DC maturation, start T cell clone expansion, start adaptive antitumor efficiency, and improve anti-PD-1 efficiency, thus generating obvious immune memory and prolonging survival time. The multi-response system combines chemotherapy with photodynamic therapy to improve the effect of immune checkpoint-blocked therapy, which has very broad application prospects.

## 5. The Clinical Studies Based on Nanocarriers for TCM Applications

With the continuous development of nanotechnology, there are now some nanocarriers applied in clinical antitumor therapy by loading active ingredients of TCM (Table 2). For example, the vincristine liposome Marqibo^®^ is a vincristine liposome injection produced by Talon Therapeutics, Inc. in the U.S., which was approved by the FDA in 2012. The encapsulation of vincristine into liposomes reduces its severe neurotoxicity and gastrointestinal toxicity and is also able to increase the circulation time of vincristine in the body and release it slowly at the vascular site of the tumor (VCR leaks easily and releases quickly) [155]. Paclitaxel liposomes for injection are used for first-line chemotherapy of ovarian cancer and later for the treatment of metastatic ovarian cancer. Liposomes loaded with doxorubicin with transferrin ligand modification (MBP-Y004) developed by Mebiopharm Ltd. (Tokyo, Japan) are also in preclinical studies. Nab-paclitaxel (Abraxane) is an FDA-approved albumin nano-delivery system used clinically for the treatment of breast, lung, and pancreatic cancers [156,157]. The binding of paclitaxel to albumin in the nanoparticles alters the way the drug is transported between cells, and therefore the recommended dosage and dosing schedule for albumin-bound paclitaxel is different from that of conventional paclitaxel formulations. In addition, the formation of micelles with block copolymer materials as drug-delivery carriers was first proposed by Yokoyama in 1992 and has been developed into a drug-delivery technology, which is attracting more and more attention as a number of antitumor drugs have entered the clinical trial stage. At prese”t, The polymeric micelle paclitaxel (Genexol-PM) has been approved and marketed in China for the first-line treatment of epidermal growth factor receptor (EGFR) mutation-negative and anaplastic lymphoma kinase (ALK)-negative, non-surgically resectable, locally advanced or metastatic non-small-cell lung cancer (NSCLC) and has become the first domestically marketed paclitaxel micellar formulation [158,159]. NK105 is a polymer micelle encapsulating paclitaxel developed by Kataoka’s group and NanoCarrier as early as 1990. The polymer material used is a poly(ethylene glycol)-poly(aspartic acid) block copolymer (mPEG-b-P(Asp)) to poly(aspartic acid) form the hydrophobicity of the micelle and amic acid to form the hydrophobic core of the micelles, increasing the drug loading to 23% with a particle size of 85 nm, which has now completed phase III clinical trials [160].

## 6. Limitations of Nanocarriers for TCM Applications and the Potential Toxicity

Although various types of nanoparticles with different functions have been developed, there are still fewer nano-delivery systems that can be applied to clinical treatment. This is primarily due to the following points: (1) Most nanocarriers are complicated and costly to prepare, and therefore not easy to industrialize, which hinders their large-scale clinical application. (2) Most nano-delivery systems have poor stability and are prone to aggregation or drug leakage during carrier storage and transportation, which affects the clinical therapeutic efficacy. (3) Nano-delivery systems utilizing TMEs are still in their infancy, and determining how to further overcome the complex biological barriers in TME remains a challenge in drug delivery. For example, EPR is dependent on the degree of tumor vascularization and angiogenesis, nanoparticle accumulation in different tumors is heterogeneous, and most nano-delivery systems still have poor delivery efficiency to solid tumors. Although active targeting can further improve tumor accumulation, plasma proteins may adsorb on the surface of nanoparticles to form protein crowns when the delivery system interacts with the bloodstream, which may reduce or even eliminate the specificity of ligands to the corresponding receptors, or specific recognition with the corresponding receptors, which has an impact on both the active targeting and cellular uptake of the nano-delivery system. Therefore, there is uncertainty about the in vivo application of nano-delivery systems in clinics. (4) Although many mouse models have been developed to study human tumors, human tumors differ from mice in many aspects, such as tumor stromal cells, metabolic rates, pharmacokinetics, etc., and the use of xenograft tumor models to test drug responses may not recapitulate clinical realities in patients. Therefore, further understanding of the microenvironment of human tumors is crucial for the application of nanomedicine in clinics. (5) The safety of nanocarriers has been a hot topic of widespread concern. In order to improve the stability of nanocarriers or to confer their functionality, certain toxic excipients or non-biodegradable nanomaterials may be used in the preparation process, which in turn may cause certain adverse reactions to the human body and affect clinical treatment. Therefore, long-term toxicity studies of nano-delivery systems are necessary. (6) Critical evaluation of sterility and endotoxin in nanoparticles and nanoformulations is of great importance because most nanoparticles are used for the intravenous treatment of cancer, and these factors are neglected in the early stages of development. (7) Currently, drugs in clinical applications are primarily based on traditional chemotherapeutic drugs, and research on active ingredients of traditional Chinese medicines is still relatively scarce. Overall, there are still many challenges for nano-delivery systems to fully realize clinical translation. Therefore, in the future, the development of novel nano-delivery systems should be based on more in-depth studies on the nature of the anti-tumor active ingredients of Chinese medicine, designing delivery systems with different functions according to their deficiencies and developing more precise delivery strategies. At the same time, more attention should be paid to the in vivo study of the delivery system to lay the foundation for clinical research.

## 7. Conclusions

Clinical scientists are increasingly interested in TCM, one of the most important treasures of Chinese traditional culture. TCM’s antitumor-active ingredients are becoming increasingly prominent in tumor therapy as isolation techniques and drug action mechanisms are explored. Compared to synthetic drugs, they have good efficacy against multiple targets and lower toxicity. However, the poor water solubility and stability, poor tissue permeability, rapid in vivo clearance, and short half-life of many antitumor active ingredients of TCM, as well as their limited accumulation in target tissues, have limited widespread clinical applications and more in-depth studies. Nanotechnology-based drug-delivery systems improve many of the limitations of the antitumor active ingredients of TCM by enhancing tissue targeting, alleviating the side effects caused by off-targeting, and improving in vivo stability and local bioavailability.

Currently, the most commonly used nano-delivery systems for the antitumor active ingredients of TCM primarily include package delivery systems and covalent binding pro-drug systems. The encapsulated drug-delivery system introduces the active ingredient into the nanostructure via physical encapsulation, which is a simple drug-loading method, and the encapsulation rate and amount can be adjusted. However, the encapsulated active ingredient may leak or be released early during the long circulation process in vivo. In contrast, a covalently bound pro-drug system can prevent drug leakage during the body’s circulation and maintain drug stability and improve the pharmacokinetic characteristics of the active ingredient. However, the covalent chain between the drug and the carrier in this drug-delivery system is not easily broken, and sometimes the active drug is not fully released when it reaches the tumor site, reducing the drug efficacy. The design of multiple environment-responsive delivery systems, in which two or more stimulus-responsive units are introduced into a single pro-drug-delivery system, is expected to control the full release of the drug and thus improve the efficacy. Moreover, most TCM nano-delivery systems for antitumor treatment use a single active ingredient, and insufficient research on multi-component systems has limited the clinical efficacy and application of TCM. With the continuous improvement of the theories, research methods, and technical levels related to TCM and nano-delivery systems, more attention should be paid to the development of multi-environmentally responsive nano-delivery systems carrying multiple components, higher tissue targeting, and better biocompatibility and stability.

## Figures and Tables

**Table 1:** Common lipid-based nano-delivery systems used to encapsulate active antitumor ingredients from TCM.

Carrier Type	Material	Drug	Tumor Type	Ref.
Liposome	Cholesterol (CHOL) Stearylamine Soy lecithin poly (ε-caprolactone)	PTX	Breast Cancer	[60]
	PolyethyleneGlycol (PEG) CHOL Soy lecithin (SPC)	CUR SN38	Lung Cancer	[61]
	Poly(ethyleneglycol)-poly(lacticacid) (PEG-PLA) Dipalmitoylphosphatidylcholine (DPPC)	CPT	Colon Cancer	[62]
	SPC CHOL Mycoplasma Membrane	Podophyllotoxin (POD)	Breast Cancer	[63]
	1,2-distearoyl-sn-glycero-3-phosphoethanolamine-N-[methoxy(polyethylene glycol)-2000] (DSPE-mPEG2000) CHOL Egg yolk lecithin	Oxaliplatin PTX	Ovarian cancer	[64]
SLNs	Chitosan Polyvinyl alcohol MTT(3-(4,5-dimethylthiazol-2-yl)-2,5-diphenyltetrazolium bromide)	Docetaxel	Melanoma Colorectal Cancer	[65]
	Stearic acid SPC	PTX	Ovarian Cancer	[66]
	HSPC 1,2-distearoyl-sn-glycero-3-phosphoethanolamine-N-[methoxy(polyethylene glycol)-2000] (DSPE-PEG2000)	CUR PTX	Lung Cancer	[42]
	Alpha-tocopherol polyethylene glycol 1000 succinate (TPGS) Stearic acid EPC	Resveratrol (Res)	Breast Cancer	[67]
NLCs	1,2-Distearoyl-sn-glycero-3-phosphoethanolamine-N-[maleimide(polyethylene glycol2000)](DSPE-PEG2000-Mal)	Docetaxel (DTX) Tariquidar (TRQ)	Breast Cancer	[68]
	Oleic Stearic acids	Curcumin (CRN)	Prostate Cancer	[50]
	Phospholipon PEG 4000 monostearate Octadecylamine	Doxorubicin (DT) Curcumin (CR)	Non-small Cell Lung Cancer	[69]
	Myristyl myristate (MM), Miglyol 812^®^ (MG)	Doxorubicin (DTX) Lidocaine (LDC)	Melanoma	[70]
Microemulsion and self-micro emulsion drug delivery system	Fumed colloidal silica Vitamin E a-Tocopherol polyethylene glycol succinate (TPGS) Gelucire^®^ Capryol^®^90	PTX	Breast Cancer	[71]
	Polysorbate 80	CPT	Colorectal Cancer	[72]
	Enoxaparin (Enox) PeceolTM (glyceryl monooleate) Cremophor EL (polyoxyl-35 castor oil) Labrafil M 1944 (oleoyl polyoxyl-6 glycerides) propylene glycol (PG)	DTX	Non-small Cell Lung Cancer	[73]

**Table 2:** The general situation of application of various nanocarriers.

Nanocarriers	First Development	Advantages	Limitations	Improvement Methods	Clinical Application	Ref.
Liposome	1960s	Biologically inert Biodegradable Biocompatible Low inherent toxic	Thermodynamically unstable systems Rapid clearance from the bloodstream Drug leakage	Modification of natural or synthetic polymeric molecules on the surface of liposomes Develop SLNs	Doxil^®^ Myocet^®^ Marqibo^®^ Lipoplatin^TM^ EndoTAG-1	[35,36,37]
Solid Lipid Nanoparticles	1990s	Biocompatibility Slow water absorption Greater stability Low sensitivity to erosion	Temperature during Preparation affects stability	Drying of SLNs to powder form for storage Develop NLCs	Mucosolvan Retard Nanobase	[43,44]
Nanostructured lipid carriers	1999/2000s	Increased drug-loading capacity Low toxicity More stable in storage Reduced drug leakage	Toxicity studies required for High concentrations of NLCs	Optimizing the physicochemical properties of drug and lipid components	_	[53,54]
Microemulsion and self-micro emulsion drug delivery system	1970s	Multiple routes of administration Simple preparation process Easy to industrialize Low viscosity	Examine its safety	Searching for efficient and low-toxicity emulsifiers and emulsifiers	_	[58,59]
Polymer micelles	1992s	Easy retouching Structurally stable Hydrophilic outer layer to avoid macrophage phagocytosis	Only insoluble drugs can be loaded	Optimized micelle structure	Genexol^®^PM NK105 Paclical^®^PM	[78,79]
Polymer nanoparticles	1992s	Small size High specific surface area	Poisonous Particle aggregation	Optimizing the composition of polymer nanoparticles	_	[87]
Dendrimers	1985s	Load multiple types of drugs Increase drug solubility Commonly used for nucleic acid and small molecule delivery	Toxicity Non-degradability	Selection of biocompatible or biodegradable materials Surface modification of dendrimers	VivaGel	[90]
Gold nanoparticles	1951s	Easy surface finishing High biosafety Electrochemical characterization	High preparation costs Easy to be oxidized	Modification of the structure of NPs	_	[102,103]
Mesoporous silica nanoparticles	1990s	Many holes Large surface area Easy to modify	Limited drug-carrying capacity Leakage of drugs Slower metabolism	Development of HMSNs Development of degradable MSNs Avoiding drug leakage by encapsulating MSNs with membranes or plugging pores	_	[107,110,111]
Metal-Organic Frame	1990s	Large specific surface area Strong adsorption performance Early cancer diagnosis in vitro	Poor stability Complex synthesis process Difficulties in industrial production	Improved synthesis of MOFs	HKUST-1	[161,162]

## Data Availability

Not applicable.
